# Should the Current Zero HLA ABDR-Mismatch Priority in Kidney Allocation System Continue? A Critical Appraisal

**DOI:** 10.34067/KID.0000001132

**Published:** 2026-01-13

**Authors:** Douglas Keith, Elizabeth Lessmann

**Affiliations:** Ascension Sacred Heart Kidney Transplant Program, Pensacola, Florida

**Keywords:** transplant outcomes, deceased donor organ transplantation

## Abstract

**Key Points:**

- Zero HLA ABDR mismatch priority in the Kidney Allocation System is highly biased against minority candidates.
- Zero HLA ABDR mismatch priority shunts 2.5% of O donors to nonidentical blood types disadvantaging O recipients.
- Zero HLA ABDR-mismatch priority recipients have substantially shorter dialysis exposure before transplant than nonpriority recipients.

**Background:**

In the Kidney Allocation System (KAS), adult kidney candidates who are a zero HLA ABDR-mismatch with a potential donor are given priority in the match run over nonzero HLA ABDR-mismatch candidates. Furthermore, KAS allows for the shunting of kidneys to zero-mismatch candidates across compatible ABO blood types, potentially disadvantaging O candidates who have longer waiting times. This study was undertaken to determine who is receiving zero HLA ABDR-mismatch kidneys, the number of donors shunted to nonidentical ABO recipients, the effect on dialysis exposure before transplant, and the current patient and graft outcomes.

**Methods:**

All adult deceased donor kidney recipients since the start of KAS on December 4, 2014, were included in the study. Multiorgan transplants with a kidney, 98%–100% calculated panel reactive antibodies (CPRAs), previous living donors, pediatric, medically urgent, and safety net transplants were excluded. Patients were considered a zero-mismatch allocation if they received a zero HLA ABDR-mismatch kidney and had an allocation CPRA <98%. One hundred twenty-two thousand nine hundred and fifty-one adult deceased donor kidney transplants were in the study population. Six thousand two hundred twenty-eight, or 5.1%, were zero HLA ABDR-mismatch recipients.

**Results:**

The zero HLA ABDR-mismatch recipients were predominantly of the White race, female, had lower allocation CPRAs, much shorter dialysis exposure, and were more likely to be retransplant recipients. One thousand four hundred and forty-one blood type O donors were shunted to other ABO groups, or 2.46% of O donors. Cox analysis of graft and patient survival showed that zero HLA ABDR-mismatch recipients had a small graft survival advantage (0.898, 0.834 to 0.967, *P* = 0.004) and no patient survival advantage (0.947, 0.865 to 1.037, *P* = 0.239). The adjusted graft survival at 7 years was 67% (zero HLA-ABDR mismatch) versus 65% (nonzero HLA-ABDR mismatch).

**Conclusions:**

The current zero HLA ABDR-mismatch priority is highly biased against racial minority recipients, shunts a sizable number of O donors to non-O recipients, only makes a small improvement to organ utility, and should be removed from the allocation priority list.

## Visual Abstract

**Figure:**
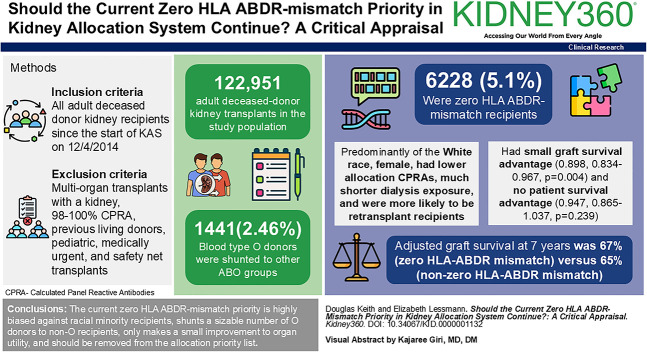

## Introduction

For patients with ESKD, a kidney transplant remains the best treatment for those candidates who qualify. Unfortunately, the demand for kidney transplants remains significantly greater than the supply of transplantable kidneys, resulting in a large candidate waiting list for kidney transplants approaching 100,000 people in the United States.^1^ To provide fair and equitable rules for the allocation of the available transplant organs, the Department of Health and Human Services established the Final Rule on March 16, 2000, which nationalized the organ allocation system and established the Organ Procurement and Transplantation Network (OPTN) to oversee, develop, and implement national rules for organ allocation.^2^ The development of the Kidney Allocation System (KAS) involves multiple stakeholders in a committee-based process with public feedback. The development of allocation rules is an iterative process that requires ongoing modification as new data on the allocation system's performance become available and as new stresses or demands on the system are identified. Although science and ethics provide guardrails, allocation development is ultimately a political process, constrained by organ shortages and requiring trade-offs among competing stakeholders.

In the current KAS, a hierarchy of priority groups determines how candidates will be prioritized for allocation. For instance, candidates who qualify for a multiorgan transplant with a kidney have the highest priority when an organ offer is available in the current system. Priority groups are ranked above where the majority of adult kidney-alone candidates receive offers. The priority groups include pediatric recipients who are more detrimentally affected by delays in transplant or previous living donors who have developed kidney failure and now require a kidney transplant to acknowledge and lessen the effect of their previous donation. Most of the other priority groups are designed to improve equity of access or improve the utility of organ use. The zero HLA ABDR-mismatch priority group allows candidates without HLA mismatches to donor HLA A, B, and DR to receive offers before nonzero HLA ABDR-mismatch candidates, based on the greater utility of organ use (HLA C, DQ, and DP are not considered). In the case of utility-based priority groups, three questions need to be considered: first, how much does the priority improve utility and based on what metrics (*i.e*., graft survival versus patient survival); second, how does the priority impact candidates and recipients that are unlikely to benefit from the priority; and third, which candidates are likely to benefit and which candidates are likely not to benefit or how discriminatory is the rule?

Although the ranking of priority groups varies some based on the four Kidney Donor Profile Index (KDPI) lanes of the donor and location of the recipient to the donor, the basic allocation sequence of the priority groups is as follows: multiorgan with a kidney candidates; 100% calculated panel reactive antibodies (CPRAs) candidates, candidates who previously were living donors, pediatric candidates, medically urgent candidates, 99%–98% CPRA candidates, zero HLA ABDR-mismatch candidates, heart, lung, and liver safety net candidates (KDPI 21% or higher), A2 and A2B donors to B candidates, and finally the remaining adult kidney-alone candidates. Currently, 30.9% of kidneys are allocated to priority groups before the main match run for most adult kidney-alone candidates. Adult candidates with a zero HLA ABDR-mismatch to a potential donor are prioritized in match runs over non–zero-mismatch candidates, despite older analyses of the allocation system, indicating that the zero-mismatch system was highly biased against minority candidates.^3^ The inclusion of this priority was based on better long-term graft survival and, therefore, greater utility of the system under this policy.^4^ Furthermore, the KAS zero HLA ABDR-mismatch priority allows for the shunting of kidneys to zero-mismatch candidates across compatible ABO blood types, potentially disadvantaging O candidates who already have longer waiting times than A or AB candidates.

Over time, the KAS in the United States has reduced the emphasis on HLA mismatching due to its inherent bias in favor of the dominant racial and ethnic group in the donor and recipient pools, White patients, and evidence that class one HLA antigens have less effect on outcomes.^5–8^ The two remaining HLA-mismatch features in KAS are the zero HLA ABDR-mismatch priority group and the HLA DR-mismatch points in the rank ordering of match-run candidates. There is evidence that the effect of HLA mismatching is decreasing due to better immunosuppression and histocompatibility testing.^9^ Furthermore, zero HLA ABDR-mismatch priority and HLA DR-mismatch scoring allow qualifying candidates to jump ahead of other candidates with longer waiting times and dialysis exposure before transplant in the offer sequence. Multiple studies from different countries indicate that dialysis exposure before transplant has a significant effect on graft and patient outcomes.^10–14^ Therefore, the current policy may disadvantage candidates who are less likely to qualify for zero HLA ABDR-mismatched donors. The purpose of this study was to determine who is receiving these zero HLA ABDR-mismatch kidneys in KAS, how many kidneys are shunted to nonidentical but compatible blood types, the policy's effect on dialysis exposure prior to transplant, and whether the current patient and graft outcomes justify the priority.

## Methods

The study was a retrospective observational analysis of all adult deceased-donor kidney-alone recipients in the Scientific Registry of Transplant Recipients (SRTR) database since the start of KAS on December 4, 2014. The end date of the dataset was February 6, 2024. Patients were considered to have received a zero HLA ABDR-mismatch kidney if their allocation CPRA was not 98%–100%, since these highly sensitized candidates have priority over less sensitized zero HLA ABDR-mismatch candidates. Multiorgan with a kidney, pediatric, 98%–100% CPRA, prior living donors, and medically urgent recipients were excluded because these groups are allocated ahead of zero HLA ABDR-mismatch candidates. Safety-net transplants were excluded because they have very short waiting times and would bias the results. A2 and A2B donors to B recipients were included in the analysis, since they were added to improve transplant rates in B recipients (equity-based priority group) and allocated after the zero HLA ABDR-mismatch priority group. Basic demographic characteristics of the donor and recipients were determined including recipient age, SRTR-determined race and ethnicity, gender, HLA mismatch, cause of renal failure (diabetes mellitus, hypertension, GN, polycystic kidney disease, and other), duration of dialysis before transplant, cold ischemia time, previous transplant recipient, allocation CPRA, and ABO type. The donor variables included KDPI 2022 (age, height, weight, cause of death, cerebrovascular accident, diabetes mellitus, hypertension, donor after cardiac death, and donor creatinine), SRTR-determined race, gender, and ABO type.

IBM Statistical Product and Service Solutions software version 29.0.2.0 was used for statistical analysis. Categorical variables were expressed as percentages, and chi-squared goodness of fit was used to determine the statistical significance. Continuous variables that were not normally distributed were reported as medians with interquartile ranges, and the Mann–Whitney *U* test was used to determine statistical significance. Continuous variables with normal distributions were reported as mean±SD, and ANOVA was used to assess significance. Cox proportional hazards modeling was used to evaluate the independent effect of a zero HLA ABDR-mismatch on patient and graft survival. Data were censored if the recipient was lost to follow-up or at the end date of their follow-up without an event, either death or graft loss. Analysis of graft survival included graft loss due to death with graft function. A *P* value < 0.05 was considered significant. To assess the time-independence of the variables used in the Cox analysis, Schoenfeld residuals were examined.

In total, 2.4% of cases had missing values (3055 of 122,951 cases). Only two variables had missing data: donor creatinine (needed to calculate KDPI 2022), missing in 1.2% of cases, and cold ischemia time, missing in 2.2% of cases. The percentage of missing data between the two groups, zero versus nonzero HLA ABDR-mismatch recipients, was nearly identical: donor creatinine 1.2% and 1.2%, respectively, and cold ischemia time 2.2% and 2.1%, respectively. Multiple imputation was used to assess the effect of missing data on estimates of patient and graft survival. Five imputations were performed for each missing variable using the Markov Chain Monte Carlo method in Statistical Product and Service Solutions. The results of the Cox analysis of the imputed data file are shown in the Supplemental Data.

This study used data from the SRTR. The SRTR data system includes data on all donor, waitlisted candidates, and transplant recipients in the United States, submitted by the members of the OPTN. The Health Resources and Services Administration, US Department of Health and Human Services provides oversight to the activities of the OPTN and SRTR contractors.

## Results

**Figure 1:**
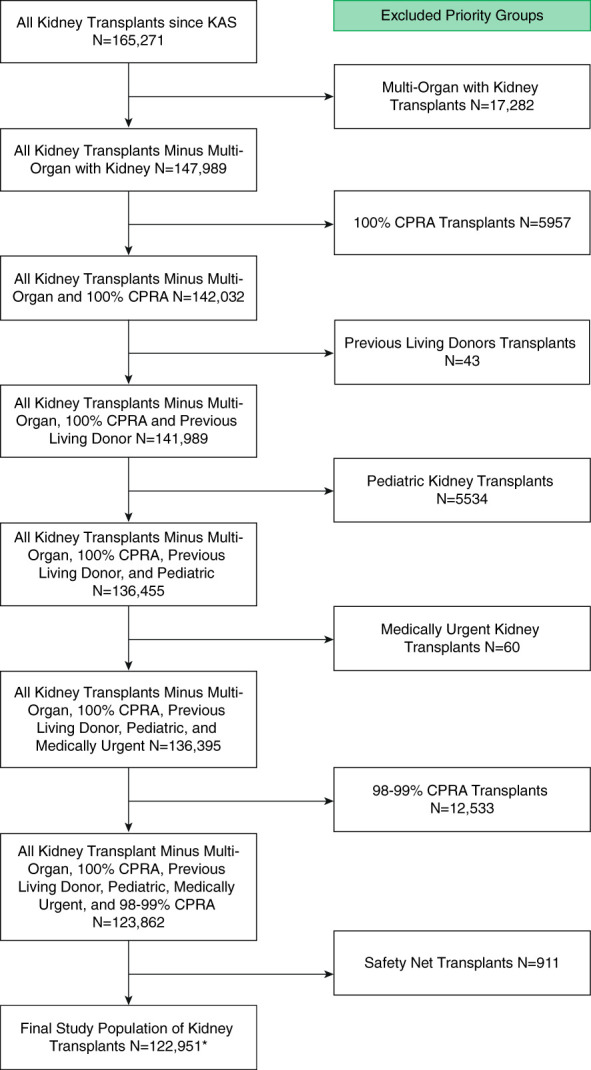
**Study population and exclusions.** *This includes A2 and A2B to B recipients *n*=2563. CPRA, calculated panel reactive antibody; KAS, Kidney Allocation System.

Figure 1 shows the breakdown of the excluded patients. A total of 165,271 deceased donor kidney transplants were performed during the study period. After excluding multiorgan transplants with a kidney, 100% CPRA, prior living donor, pediatric, medically urgent, 98%–99% CPRA, and safety net transplants, 122,951 adult deceased-donor kidney transplants constituted the study population. Six thousand two hundred and twenty-eight (5.1%) were zero HLA ABDR-mismatch recipients but not highly sensitized (allocation CPRA <98%). A2 and A2B to B transplants accounted for 2563 transplants during KAS and are included in the study population. During KAS, 30.9% of transplanted kidneys were allocated to one of the priority groups, ahead of the main adult kidney-alone nonzero HLA ABDR-mismatch match run candidates. Table 1 presents recipient characteristics by HLA ABDR-mismatch allocation status (zero or nonzero). The zero HLA ABDR-mismatch recipients were more likely to be White recipients, female, had lower allocation CPRAs, had much shorter dialysis exposure, and were more likely to be retransplant recipients. The recipient ABO showed more A recipients and fewer O, AB, and B recipients in the zero HLA ABDR-mismatch cohort. Conversely, the donor ABO distribution showed that O donor kidneys were much more prevalent, and A, AB, and B kidneys were less prevalent donor ABO types in the zero HLA ABDR-mismatch cohort, reflecting the shunting of O kidneys to nonidentical blood types. The median KDPI 2022 was similar between recipients of zero and nonzero HLA ABDR-mismatches.

**Table 1:** Characteristics of the study population

Characteristics of Transplants	Zero HLA Mismatch Recipient (*n*=6228)	Nonzero Mismatch Recipient (*n*=116,723)	*P* Value
Recipient median age in years with quartiles	54 (43, 63)	55 (44, 64)	<0.001
Recipient gender male	48.9% (3045)	60.6% (70,785)	<0.001
**Recipient race and ethnicity**			<0.001
Asian	2.9% (181)	8% (9328)	
Black	10.6% (663)	36.6% 42,747)	
Latino	21% (1310)	19.8% (23,082)	
Multiracial	0.7% (44)	0.8% (935)	
Native American	0.9% (53)	0.9% (1055)	
Pacific Islander	0.2% (11)	0.5% (610)	
White	63.7% (3966)	33.4% (38,966)	
**Duration of dialysis pretransplant, yr**			<0.001
Preemptive	20.5% (1274)	9.5% (11,085)	
<1	13.7% (856)	8.3% (9650)	
1–2	18.7% (1163)	10.6% (12,389)	
2–3	15.9% (988)	11.6% (13,535)	
3–4	10.1% (629)	11.6% (13,571)	
4–5	7.3% (456)	11.5% (13,435)	
5–6	4.6% (284)	10.2% (11,912)	
6–7	2.7% (170)	8.3% (9672)	
7–8	1.7% (103)	5.7% (6696)	
8–9	1.2% (73)	3.9% (4607)	
9–10	0.9% (54)	2.7% (3200)	
10+	2.9% (178)	6% (6971)	
Median time on dialysis in years with quartiles	1.85 (0.40, 3.55)	3.87 (1.70, 6.18)	<0.001
**Allocation CPRA group**			<0.001
0%–20%	53.8% (3349)	50.6% (59,071)	
21%–40%	10.7% (669)	11% (12,791)	
41%–60%	8.9% (554)	9.7% (11,267)	
61%–80%	10.3% (644)	11.4% (13,297)	
81%–90%	7.8% (485)	7.8% (9108)	
91%–97%	8.5% (527)	9.6% (11,189)	
Median CPRA with quartiles	20% (2%, 68%)	25% (3%, 71%)	<0.001
**Cause of kidney failure**			<0.001
Diabetes mellitus	28.6% (1779)	30.8% (36,006)	
GN	14.1% (876)	13.5% (15,761)	
Hypertensive nephrosclerosis	16.1% (1004)	25.4% (29,679)	
Polycystic kidney disease	30.9% (1923)	23.1% 26,988)	
Other cause	10.4% (646)	7.1% (8289)	
**Recipient ABO**			<0.001
A	39.9% (2488)	34.4% (40,122)	
AB	3.2% (199)	5.2% (6105)	
B	11.8% (738)	14.3% (16,699)	
O	45% (2803)	46.1% (53,797)	
**HLA mismatch**			<0.001
0	100% (6228)	0% (0)	
1	0% (0)	1.4% (1661)	
2	0% (0)	5.2% (6105)	
3	0% (0)	15% (17,532)	
4	0% (0)	29% (33,891)	
5	0% (0)	33.8% (39,441)	
6	0% (0)	15.5% (18,093)	
Previous organ transplant	27.3% (1701)	12.3% (14,351)	<0.001
**Donor characteristics**			
KDPI^a^			<0.001
0%–9%	3.3% (206)	4% (4667)	
10%–19%	8.1% (496)	10.6% (12,184)	
20%–29%	13.7% (843)	14% 16,182)	
30%–39%	18.1% (1116)	14.9% (17,167)	
40%–49%	17.6% (1081)	14.1% (16,209)	
50%–59%	14.6% (901)	13% (14,970)	
69%-69%	11.3% (697)	11.4% (13,103)	
70%–79%	7.7% (473)	8.8% (10,169)	
80%–89%	3.8% (235)	6.2% (7102)	
90%–100%	1.7% (107)	3.1% (3517)	
Median KDPI with quartiles	43% (29%, 59%)	44% (27%, 63%)	0.024
Donor gender male	61.2% (3811)	62.5% (72,962)	0.037
**Donor race**			<0.001
Asian	1.8% (114)	2.4% (2875)	
Black	4.5% (279)	14% (16,934)	
Multi-racial	0.3% (17)	0.4% (472)	
Native American	0.9% (55)	0.8% (951)	
Pacific Islander	0.2% (11)	0.3% (358)	
White	92.4% (5752)	82.1% (95,133)	
**Donor ABO**			<0.001
A	27.2% (1692)	37.7% (43,990)	
AB	0.5% (30)	3.8% (4385)	
B	4.2% (262)	11.9% (13,938)	
O	68.1% (4244)	46.6% (54,410)	
Donor from outside 250 mile radius or OPO	79% (4918)	41.1% (47,995)	<0.001
Mean cold ischemia time in hours with SD^a^	20.4±7.0	18.9±8.2	<0.001

CPRA, calculated panel reactive antibody; KDPI, Kidney Donor Profile Index; OPO, Organ Procurement Organization.

a Denotes two variables with missing data: cold ischemia time (missing data 2.2%) and Kidney Donor Profile Index (missing data 1.2% serum creatinine).

Table 2 presents the ABO match between the donor and recipient of zero HLA ABDR-mismatch recipients. One thousand four hundred and forty-one O blood type donors were shunted to other ABO recipients, or 2.46% of O donors during the study period. In contrast, very few A, AB, or B donors were shunted to nonidentical blood types.

**Table 2:** Zero HLA ABDR-mismatch donor and recipient ABO match

Donor ABO	Recipient ABO				Number Shunted to Nonidentical Blood Group Recipients	Total Number of Donors By ABO Group in Study	Percent of Kidney Shunted to Nonidentical Blood Group Recipients
	A	AB	B	O			
A	1611	77	4	0	81	45,682	0.18%
AB	0	27	3	0	3	4415	0.07%
B	0	31	231	0	31	14,200	0.22%
O	877	64	500	2803	1441	58,654	2.46%

**Figure 2:**
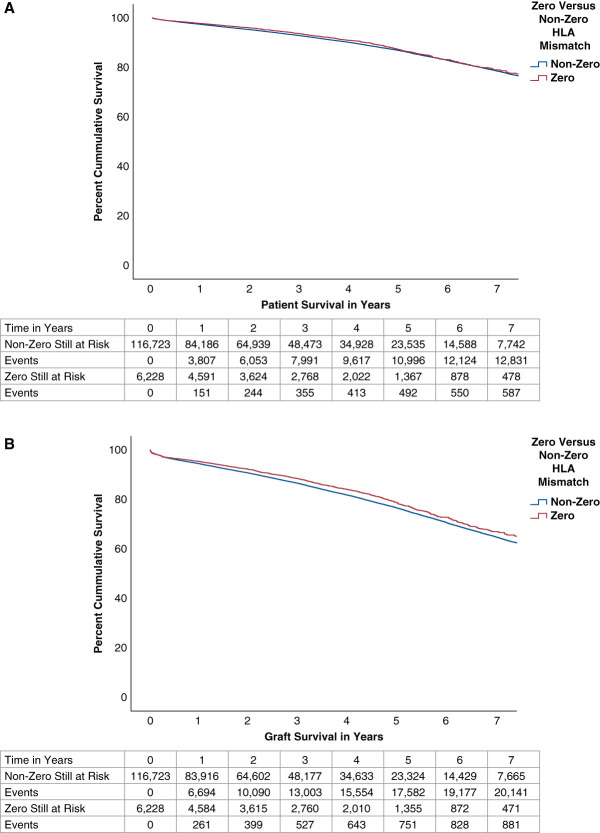
**Adjusted patient and graft survival based on zero or nonzero HLA ABDR-mismatch allocation.** (A) Patient survival. (B) Graft survival.

Figure 2 shows the adjusted patient and graft survival of kidney transplant recipients based on zero versus nonzero HLA ABDR-mismatch allocation. The adjusted graft survival at 7 years was 67% (zero HLA mismatch) versus 65% (nonzero HLA mismatch), whereas adjusted patient survival was the same for the two groups at 7 years, at 78% (Figure 2, A and B). Cox analysis of graft and patient survival adjusting for multiple covariates showed that zero HLA ABDR-mismatch recipients had a small graft survival advantage (0.898, 0.834 to 0.967, *P* = 0.004) and no patient survival advantage (0.947, 0.865 to 1.037, *P* = 0.239; see Supplemental Tables 1A and 1B). This compares with the unadjusted hazard ratio of 0.788 (0.737 to 0.841) for graft survival and 0.827 (0.783 to 0.896) for patient survival. The multiple imputation analysis of missing data did not change statistical outcomes for patient and graft survival (see Supplemental Tables 2A and 2B). Sensitivity analysis with inclusion of highly sensitized kidney-alone recipients (98%–100% allocation CPRA) in the nonzero HLA ABDR-mismatch group did not significantly alter the results, with an adjusted hazard ratio of 0.899 (0.836 to 0.967) for graft survival and an adjusted hazard ratio of 0.934 (0.859 to 1.026) for patient survival.

Table 3 presents the hazard ratios for graft loss and death based on dialysis duration before transplant. Duration of dialysis before transplant impacts both graft and patient survival and increases with increasing time on dialysis. Across the spectrum of dialysis exposure before transplant in the dataset (ranging from preemptive to 10+ years of dialysis), the magnitude of effect on outcome was similar to that observed with KDPI for graft survival and larger than that observed with KDPI for patient survival (see Supplemental Tables 1A and 1B for complete Cox analysis).

**Table 3:** Hazard ratio of graft failure and patient survival after kidney-alone transplant

Duration of Dialysis Prior to Transplant (Reference: Preemptive), yr	Hazard Ratio of Graft Failure			Hazard Ratio of Death		
	Hazard Ratio	95% Confidence Interval		Hazard Ratio	95% Confidence Interval	
		Lower	Upper		Lower	Upper
<1	1.25	1.16	1.34	1.24	1.13	1.36
1–2	1.36	1.27	1.46	1.38	1.27	1.51
2–3	1.39	1.30	1.49	1.46	1.34	1.59
3–4	1.52	1.43	1.63	1.63	1.50	1.77
4–5	1.54	1.44	1.65	1.71	1.57	1.86
5–6	1.63	1.52	1.75	1.82	1.68	1.99
6–7	1.71	1.59	1.84	1.89	1.73	2.07
7–8	1.83	1.70	1.98	2.10	1.91	2.31
8–9	1.91	1.76	2.08	2.27	2.05	2.52
9–10	2.01	1.83	2.20	2.42	2.16	2.71
10+	2.07	1.92	2.24	2.51	2.28	2.76

Other covariates in the model: recipient age, Scientific Registry of Transplant Recipients race and ethnicity, gender, ABO blood type, cause of ESKD, calculated panel reactive antibody, previous organ transplant, donor Kidney Donor Profile Index, Scientific Registry of Transplant Recipients race, gender, ABO blood type, zero versus nonzero HLA ABDR-mismatch, and cold ischemia time.

## Discussion

The current zero HLA ABDR-mismatch priority in KAS is highly biased against racial minority candidates, especially Asian and Black patients, allows zero HLA ABDR-mismatch candidates to jump ahead of candidates with longer listing times, lengthening waiting times for those candidates who do not qualify, shunts a significant number of blood type O donors to non-O recipients, and only results in a small improvement in organ utility based on patient and graft survival after transplant. The most important modifiable risk factor for transplant is dialysis exposure before transplant.^10–14^ The median time difference between recipients with zero HLA ABDR-mismatch and those with nonzero HLA ABDR-mismatch was 2 years. Zero HLA ABDR-mismatch recipients get a significant patient survival advantage due to less dialysis exposure, since zero HLA ABDR-mismatch in and of itself does not appear to improve patient survival over nonzero HLA ABDR-mismatch recipients. The zero HLA ABDR-mismatch priority functions like a lottery for donor kidneys, but the chances of winning the lottery are dependent on the frequency of the candidate's HLA types in the donor population and are not equal for all individuals on the waiting list. Unfortunately, owing to the differences in HLA frequencies in racial groups, HLA-mismatching is inherently biased in favor of the dominant racial groups in the donor and candidate population, which leads to inequitable access based on race.

With the inclusion of the A2 and A2B to B allocation in KAS, blood type O candidates will likely become the blood group with the longest waiting times. The zero HLA ABDR-mismatch priority allows donor kidneys to be shunted to nonidentical but compatible blood groups. Based on our analysis, this primarily impacts blood type O donors, shunting about 2.5% of O donor kidneys to nonidentical blood types. Given the effect of dialysis time on outcomes on the waitlist and post-transplant, this policy is difficult to justify ethically and should be abolished for at least blood type O donor kidneys.

There are several potential weaknesses of the study. Although the Cox analysis included 20 covariates (12 individual covariates plus eight for KDPI 2022), the potential for unmeasured covariates to affect the outcomes remains. While unmeasured socioeconomic and health literacy factors likely bias results toward better patient and graft survival among preemptively listed recipients, they also bias the zero HLA ABDR-mismatch group, which is predominantly White race and has a higher percentage of preemptively listed and transplanted recipients. Since the allocation priority groups are not specified in the dataset, the priority allocation group had to be inferred from the HLA-mismatch, candidate age at listing, designation of previous living donor, medically urgent status, timing of listing for a kidney after liver, heart, or lung transplant since initiation of safety-net for those organs, and allocation CPRA recorded in the dataset. It is possible that some candidates who qualified for a priority group were allocated in a different priority group or misclassified, but we believe the number is small. In total, 2.2% of cases in the dataset had missing values, which were used for the analysis of patient and graft survival. The missing data were in two variables, donor creatinine and cold ischemia time, and in both cases, the missing data were evenly distributed between the two cohorts. Although missing data could marginally change hazard ratios in the Cox analysis, the small number of missing values and the results of the multiple imputation analysis suggest that more complete data would not likely change the fundamental conclusions of the Cox analysis, namely, that zero HLA ABDR-mismatch confers a small graft survival benefit but no patient survival benefit.

The OPTN/SRTR is exploring new allocation models using simulations that would largely abandon most priority groups in favor of a continuous-scoring model for sequencing match runs.^15^ Except for multiorgan transplants with a kidney transplant, previous high-priority groups, prior living donors, medically urgent cases, pediatric recipients, and safety-net transplants for liver, lung, and heart recipients, would receive additional priority points, resulting in a higher score and higher rank in the match-run allocation sequence. Instead of high CPRA priority groups, candidates with a CPRA would receive additional points calculated on an exponential scale that mirrors the probability of a suitable offer. The published models scored only HLA-DR mismatches and did not include HLA-A or HLA-B mismatches. In that respect, it did not have a zero HLA ABDR-mismatch score. The weighting of factors can be adjusted to achieve desired outcomes, such as improved transplant longevity in the population transplanted or reduced kidney shipments. The advantage of the continuous models is that the previous KAS priority groups were ranked equally rather than in a hierarchy from first to last and that other factors in the ranking algorithm determine a candidate's offer sequence among the priority groups. In other words, the priority groups would compete directly with one another. It also allows nonpriority candidates to compete for offers based on their scores, for instance, if they have accumulated significant waiting time.

The disadvantage of continuous-scoring models is that they are highly complex, and their function depends on the weighting of each element. In our opinion, the OPTN algorithms greatly underweigh the effect of dialysis exposure as a factor influencing outcomes relative to other factors, such as HLA DR mismatching (zero HLA DR-mismatch receives one point, while a year of waiting receives only 0.1 point in the model, even though they have almost equivalent effect on graft survival). The various versions of the models are designed to maximize downstream events such as transplant longevity or efficiency in organ distribution, and do not actually maximize equity of access from the waitlist. Depending on the weighting of elements, the system would still allow some patients to bypass others with longer waiting times. Although the published simulations did not include an HLA ABDR-mismatch priority, the models still include HLA DR-mismatching, which remains biased in favor of the dominant racial and ethnic group in the donor and recipient populations, White candidates.^16^ Basing allocation on eplet mismatching rather than HLA antigen mismatching appears to lessen the racial disparity but does not entirely eliminate the bias and adds another level of complexity to the allocation system.^17^ Finally, given the complexity of the allocation algorithm, it may be more challenging to understand how different subgroups within the listed population are allocated and to what extent the system achieves more equitable access.

In the setting of a kidney organ shortage, allocation policy development in kidney transplantation involves trade-offs among competing candidate groups (*e.g*., multiorgan transplants with a kidney, kidney-alone transplants in adults, young versus older adults, or pediatric candidates). Utilitarian and equity ethics are the intellectual underpinnings of allocation policy. Different priority groups in the priority system are based on these ethical principles. For instance, priority for highly sensitized and A2 and A2B to B candidates is based on improving equitable access to transplantation. By contrast, zero HLA ABDR-mismatch priority and low KDPI kidneys to candidates projected to live longer is based on the improved utility of organ use. The benefit of better utility of organ use is a reduction in retransplants, which improves access to transplant for all candidates. Currently, 10.8% of the waitlist is retransplant candidates.^15^ The benefits of better HLA mismatching are improved graft survival (decreased need for retransplantation)^4^ and less sensitization if the graft fails (improving the chances of retransplantation).^16–20^ However, only a small number of adult transplants are listed for retransplantation, primarily younger adult recipients.^20^ Most adult recipients receive only one kidney transplant during their lifetime. Zero HLA ABDR-mismatch recipients had a similar age distribution to nonzero HLA ABDR-mismatch recipients, indicating that any potential reduction in sensitization after graft failure in younger adult patients deemed candidates for retransplantation would be small. The 2% difference in graft survival at 7 years means that approximately 125 graft failures were avoided (2% of 6228 transplants) with the zero HLA ABDR-mismatch priority. If 50% of graft failures were due to the death of the patient, only 63 patients would be potentially eligible for relisting. Presumably, only a fraction of that number would still be healthy and young enough to undergo retransplantation. Therefore, a policy that increases inequity in allocation to improve the potential utility of organ use for a small minority of retransplant candidates who may experience less sensitization with a zero HLA ABDR-mismatch primary transplant is difficult to support.

Currently, in adult kidney-alone candidates without a living donor, only about 55% of waitlisted candidates will receive a deceased donor kidney transplant.^21^ The other 45% of candidates will either die or be removed from the waitlist. The longer a candidate waits, the more likely this outcome will occur. Similarly, dialysis duration is by far the most important modifiable risk factor for reduced patient and graft survival post-transplant.^10–14^ Because of the effect of time on dialysis on both waitlist outcomes and post-transplant graft and patient survival, utility-based allocation policies that improve some recipient outcomes but lead to inequity in access probably do not improve overall utility of the system since longer waits for nonpriority candidates and poorer outcomes associated with the increase in dialysis exposure may offset the gains made in candidates who receive a benefit from a priority allocation. In general, the effect of the zero HLA ABDR-mismatch allocation policy has focused on downstream outcomes for recipients but has ignored the upstream and downstream impacts on candidates on the waiting list and recipients who are unlikely to benefit from this allocation priority. A more comprehensive accounting of the effect may show that more harm is done to the population than benefit to those who receive a kidney with a zero HLA ABDR-mismatch.

Kidney allocation is a zero-sum game. A kidney given to one individual will result in others waiting longer for a transplant or dying without a transplant. Given the effect of dialysis duration on the competing outcome of death and delisting on the waitlist and patient survival after transplant, the guiding principle of adult kidney allocation policy should be to equitably distribute the risk of waiting on dialysis for a kidney transplant. Graft survival should be of secondary importance only in the development of allocation policy. Since the zero HLA ABDR-mismatch priority does not improve patient survival and increases inequity in access to transplantation, we believe that this priority group should be removed from KAS.

## Supplementary Material

## Data Availability

Original data generated for the study will be made available upon reasonable request to the corresponding author. Data Type: Health Care Data. Reason for Restricted Access: The data used for this analysis are available from the SRTR.

## Declarative Statements

The clinical and research activities being reported are consistent with the Principles of the Declaration of Istanbul as outlined in the Declaration of Istanbul on Organ Trafficking and Transplant Tourism.

## Supplemental Material

Supplemental Table 1A. Full Cox analysis of graft survival.

Supplemental Table 1B. Full Cox analysis of patient survival.

Supplemental Table 2A. Multiple imputation results for graft survival.

Supplemental Table 2B. Multiple imputation results for patient survival.

Supplemental Table 3A. Subgroup analysis of zero HLA ABDR-mismatch compared with nonzero HLA ABDR mismatch for graft survival by recipient SRTR race and ethnicity for White, Black, and Latino recipients.

Supplemental Table 3B. Subgroup analysis of zero HLA ABDR-mismatch compared with nonzero HLA ABDR mismatch for patient survival by recipient SRTR race and ethnicity for White, Black, and Latino recipients.

Supplemental Table 4A. Subgroup analysis of zero HLA ABDR-mismatch compared with nonzero HLA ABDR mismatch for graft survival by recipient ABO type.

Supplemental Table 4B. Subgroup analysis of zero HLA ABDR-mismatch compared with nonzero HLA ABDR mismatch for patient survival by recipient ABO type.
