# Supplementary material for: Should the Current Zero HLA ABDR-Mismatch Priority in Kidney Allocation System Continue? A Critical Appraisal
Source: Kidney360. 2026 Jan 13;7(5):1118–27. doi: 10.34067/KID.0000001132 (PMC13229415; doi:10.34067/KID.0000001132)
Supplement: Supplementary file 2 [file kidney360-7-1118-s002.pdf]

Supplemental Data:

Table 1A and B: Full Cox Analysis of Graft and Patient Survival

Table 1A: Graft Survival

	Hazard Ratio of Graft Failure			P value
	Hazard Ratio	95% Confidence Interval		
		Lower	Upper	
Recipient Characteristics				
Recipient Age Group (Reference: 18-24 Years Old)				
25-29	0.664	0.578	0.761	<.001
30-34	0.550	0.482	0.628	<.001
35-39	0.548	0.483	0.623	<.001
40-44	0.547	0.483	0.620	<.001
45-49	0.558	0.494	0.631	<.001
50-54	0.595	0.527	0.671	<.001
55-59	0.658	0.584	0.742	<.001
60-64	0.816	0.725	0.919	<.001
65-69	0.957	0.850	1.078	0.47
70-74	1.138	1.007	1.285	0.038
75-79	1.305	1.137	1.497	<.001
80+	1.636	1.307	2.047	<.001
Recipient Gender (Reference Female)	1.085	1.055	1.116	<.001
Recipient Race/Ethnicity (White)				
Asian	0.647	0.608	0.688	<.001
Black	1.032	0.998	1.068	0.069
Latino	0.774	0.743	0.807	<.001
Multi-Racial	0.948	0.806	1.115	0.518
Native American	1.016	0.890	1.159	0.817
Pacific Islander	0.745	0.603	0.920	0.006
Duration of Dialysis Pre-Transplant (Reference Preemptive)				
Less than 1 Year	1.248	1.159	1.343	<.001
1 to 1.99 Years	1.362	1.271	1.459	<.001
2 to 2.99 Years	1.389	1.298	1.486	<.001
3 to 3.99 Years	1.524	1.425	1.630	<.001
4 to 4.99 Years	1.544	1.443	1.651	<.001
5 to 5.99 Years	1.629	1.521	1.745	<.001
6 to 6.99 Years	1.710	1.592	1.837	<.001
7 to 7.99 Years	1.832	1.697	1.978	<.001
8 to 8.99 Years	1.913	1.757	2.082	<.001
9 to 9.99 Years	2.007	1.829	2.202	<.001
10+ Years	2.074	1.922	2.237	<.001

Allocation CPRA Group (0-20% Reference)				
21-40%	0.998	0.955	1.044	0.942
41-60%	0.956	0.912	1.003	0.064
61-80%	0.968	0.927	1.011	0.143
81-90%	0.958	0.911	1.007	0.091
91-97%	0.931	0.889	0.976	0.003
Cause of Kidney Failure (Reference: Diabetes Mellitus)				
Glomerulonephritis	0.656	0.624	0.689	<.001
Hypertensive nephrosclerosis	0.741	0.716	0.768	<.001
Polycystic kidney disease	0.709	0.682	0.738	<.001
Other cause	0.571	0.536	0.608	<.001
Recipient ABO (Reference O)				
A	1.067	0.942	1.209	0.308
AB	1.092	0.942	1.265	0.245
B	1.056	0.923	1.208	0.428
Previous Organ Transplant (Reference: Primary Transplant)	1.179	1.132	1.228	<.001
Donor Characteristics				
KDPI (Reference: 0-9%)				
10-19%	1.011	0.921	1.111	0.813
20-29%	1.082	0.988	1.184	0.088
30-39%	1.176	1.075	1.286	<.001
40-49%	1.312	1.199	1.435	<.001
50-59%	1.492	1.363	1.632	<.001
69-69%	1.607	1.467	1.76	<.001
70-79%	1.847	1.683	2.027	<.001
80-89%	1.958	1.777	2.157	<.001
90-100%	2.25	2.021	2.504	<.001
Donor Gender (Reference: Female)	1	0.973	1.028	0.981
Donor Race (Reference: White)				
Asian	0.986	0.904	1.074	0.742
Black	1.151	1.11	1.195	<.001
Multi-Racial	0.959	0.753	1.222	0.737
Native American	0.905	0.76	1.078	0.264
Pacific Islander	1.183	0.925	1.514	0.181
Donor ABO (Reference O)				
A	0.967	0.854	1.095	0.602
AB	0.969	0.827	1.135	0.692
B	0.93	0.809	1.069	0.306
Transplant Characteristics				
Cold Ischemia Time in Hours (Reference: 0-5.99 Hours)				
6-11.99 Hours	1.024	0.947	1.106	0.553
12-17.99 Hours	1.086	1.007	1.17	0.032

18-23.99 Hours	1.123	1.041	1.211	0.003
24-29.99 Hours	1.138	1.052	1.232	0.001
30-35.99 Hours	1.235	1.129	1.351	<.001
36-41.99 Hours	1.406	1.267	1.561	<.001
42-47.99 Hours	1.369	1.189	1.576	<.001
48+ Hours	1.388	1.12	1.72	0.003
Zero HLA-Mismatch (Reference: Non-Zero HLA-Mismatch)	0.898	0.834	0.967	0.004

The hazard ratio for recipient age is U-shaped for graft survival, with the best graft survival observed in recipients aged 40-44 years. During KAS, graft survival based on race and ethnicity showed that White, Black, Multi-Racial, and Native American patients had similar graft survival, while Asian, Latino, and Pacific Islander patients had better graft survival. Female recipient gender was associated with better survival. As expected, diabetes mellitus as a cause of kidney failure had worse graft survival than other causes of renal disease. Dialysis exposure prior to transplant was associated with incrementally worsening graft survival as noted in the manuscript. Paradoxically, the allocation CPRA showed slightly improved graft outcomes in the highest CPRA group, whereas the other groups showed no effect. The recipient ABO type had no effect. Recipients who had a previous transplant had worse graft survival than primary transplants. On the donor side, KDPI had an incremental worsening effect on graft outcomes with higher KDPI values. Black race of the donor was associated with worse graft outcomes. The ABO type of donor did not affect graft outcome. Cold ischemia time was associated with progressively worse outcomes, although the hazard ratios leveled off at longer cold ischemia times.

The unadjusted hazard ratio of graft failure for zero HLAABDR mismatch versus non-zero mismatch is 0.788 (0.737-0.841). Below is the Kaplan-Meier plot of graft survival for zero HLAABDR-mismatch versus non-zero mismatch.

Figure 1. Unadjusted Kaplan Meier plot of graft survival zero HLA ABDR mismatch versus non-zero HLA mismatch.

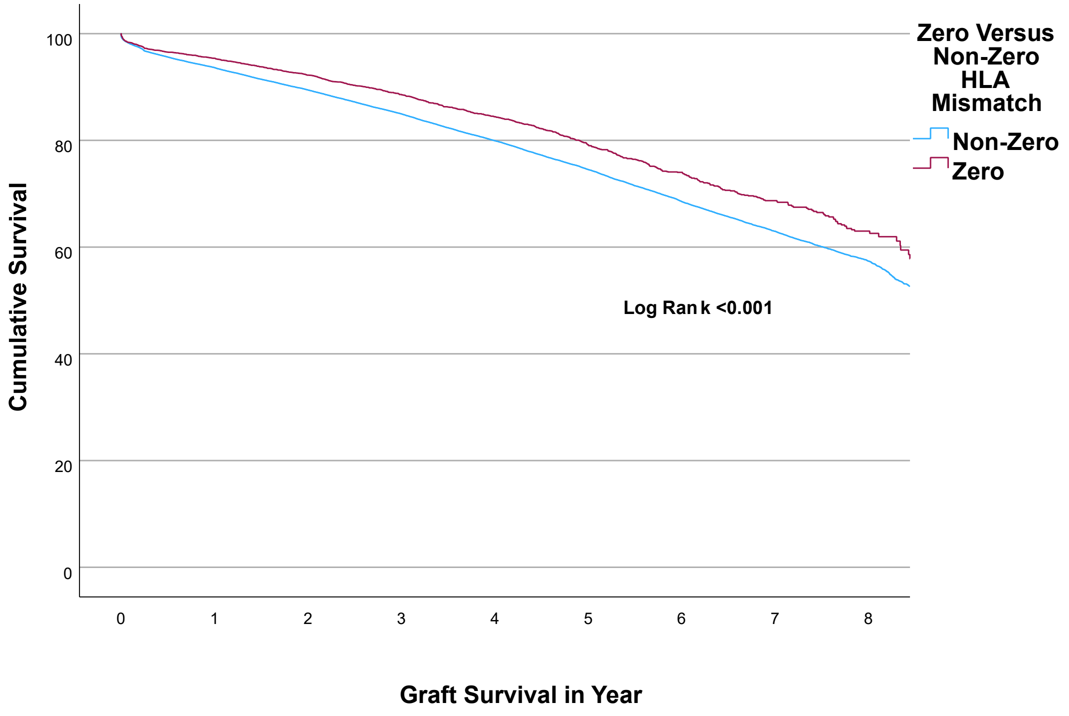

Zero Versus Non-Zero HLA Mismatch
Non-Zero
Zero
Cumulative Survival
100
80
60
40
20
0
0
1
2
3
4
5
6
7
8
Log Ran k <0.001
Graft Survival in Year

Table 1B. Patient Survival

	Hazard Ratio of Death			P value
	Hazard Ratio	95% Confidence Interval		
		Lower	Upper	
Recipient Characteristics				
Recipient Age Group (Reference: 18-24 Years Old)				
25-29	1.270	0.808	1.996	0.3
30-34	1.544	1.005	2.373	0.047
35-39	2.235	1.475	3.386	<.001
40-44	2.941	1.953	4.430	<.001
45-49	3.861	2.571	5.797	<.001
50-54	4.807	3.206	7.208	<.001
55-59	6.116	4.083	9.162	<.001
60-64	8.222	5.492	12.310	<.001
65-69	10.569	7.059	15.823	<.001
70-74	13.335	8.898	19.986	<.001
75-79	15.732	10.435	23.718	<.001
80+	21.968	14.001	34.467	<.001
Recipient Gender (Reference Female)	1.111	1.072	1.150	<.001
Recipient Race/Ethnicity (White)				
Asian	0.620	0.575	0.668	<.001
Black	0.855	0.819	0.892	<.001
Latino	0.754	0.716	0.794	<.001
Multi-Racial	0.907	0.738	1.113	0.35
Native American	1.042	0.892	1.217	0.607
Pacific Islander	0.624	0.472	0.825	<.001
Duration of Dialysis Pre-Transplant (Reference Preemptive)				
Less than 1 Year	1.238	1.129	1.359	<.001
1 to 1.99 Years	1.380	1.267	1.505	<.001
2 to 2.99 Years	1.457	1.340	1.585	<.001
3 to 3.99 Years	1.627	1.496	1.769	<.001
4 to 4.99 Years	1.706	1.569	1.855	<.001
5 to 5.99 Years	1.824	1.675	1.987	<.001
6 to 6.99 Years	1.891	1.730	2.068	<.001
7 to 7.99 Years	2.098	1.907	2.308	<.001
8 to 8.99 Years	2.272	2.046	2.524	<.001
9 to 9.99 Years	2.417	2.156	2.711	<.001
10+ Years	2.511	2.284	2.762	<.001
Allocation CPRA Group (0-20% Reference)				
21-40%	1.001	0.947	1.058	0.974

41-60%	0.970	0.915	1.028	0.303
61-80%	0.998	0.945	1.053	0.932
81-90%	0.939	0.882	1.000	0.05
91-97%	0.921	0.869	0.976	0.006
Cause of Kidney Failure (Reference: Diabetes Mellitus)				
Glomerulonephritis	0.511	0.478	0.547	<.001
Hypertensive nephrosclerosis	0.658	0.630	0.687	<.001
Polycystic kidney disease	0.625	0.595	0.657	<.001
Other cause	0.498	0.462	0.538	<.001
Recipient ABO (Reference O)				
A	1.083	0.928	1.264	0.312
AB	1.080	0.899	1.298	0.411
B	1.065	0.901	1.259	0.462
Previous Organ Transplant (Reference: Primary Transplant)	1.29	1.224	1.359	<.001
Donor Characteristics				
KDPI (Reference: 0-9%)				
10-19%	0.96	0.84	1.098	0.554
20-29%	1.016	0.895	1.154	0.803
30-39%	1.089	0.961	1.234	0.183
40-49%	1.171	1.034	1.327	0.013
50-59%	1.271	1.122	1.441	<.001
69-69%	1.294	1.14	1.469	<.001
70-79%	1.473	1.296	1.674	<.001
80-89%	1.471	1.288	1.68	<.001
90-100%	1.629	1.41	1.881	<.001
Donor Gender (Reference: Female)	1.015	0.981	1.051	0.399
Donor Race (Reference: White)				
Asian	0.991	0.891	1.103	0.87
Black	1.103	1.052	1.156	<.001
Multi-Racial	0.942	0.693	1.281	0.704
Native American	1.031	0.839	1.266	0.774
Pacific Islander	1.065	0.774	1.466	0.699
Donor ABO (Reference O)				
A	0.967	0.83	1.128	0.67
AB	1.035	0.851	1.258	0.732
B	0.945	0.795	1.124	0.524
Transplant Characteristics				
Cold Ischemia Time in Hours (Reference: 0-5.99 Hours)				
6-11.99 Hours	1.037	0.94	1.144	0.463
12-17.99 Hours	1.103	1.003	1.213	0.042
18-23.99 Hours	1.129	1.026	1.242	0.013
24-29.99 Hours	1.138	1.029	1.258	0.012

30-35.99 Hours	1.218	1.087	1.365	<.001
36-41.99 Hours	1.345	1.179	1.535	<.001
42-47.99 Hours	1.199	0.998	1.439	0.052
48+ Hours	1.16	0.869	1.548	0.315
Zero HLA-Mismatch (Reference: Non-Zero HLA-Mismatch)	0.947	0.865	1.037	0.239

Age of the recipient had the most dramatic impact on patient survival, with survival exponentially worsening as age increased. Female recipient gender had better patient survival after transplant. Whites, Multi-Racial, and Native American patients all had worse patient survival than Asian, Black, Latino, and Pacific Islander patients. Dialysis exposure before transplant showed incrementally worse patient survival with more dialysis time, as noted in the manuscript. CPRA groups showed little impact on patient survival, although the highest two CPRA groups were marginally better than the lower CPRA groups. The ABO blood type of the recipient did not affect patient survival. Recipients with diabetes mellitus as a cause of their kidney failure had worse patient survival than other causes of renal failure. Primary transplants have better survival than recipients with a previous transplant. KDPI showed incrementally worse patient survival with increasing KDPI. Black donor race was associated with worse patient survival than other donor racial groups. Donor ABO blood type did not affect outcomes. Cold ischemia time was associated with progressively worse patient outcomes as it increased, though the impact leveled off at longer cold ischemia times.

The unadjusted hazard ratio for patient survival with zero HLA ABDR-mismatch versus non-zero mismatch is 0.827 (0.783-0.896).

Figure 2. The Kaplan-Meier plot of unadjusted patient survival for zero versus non-zero HLA ABDR-mismatch.

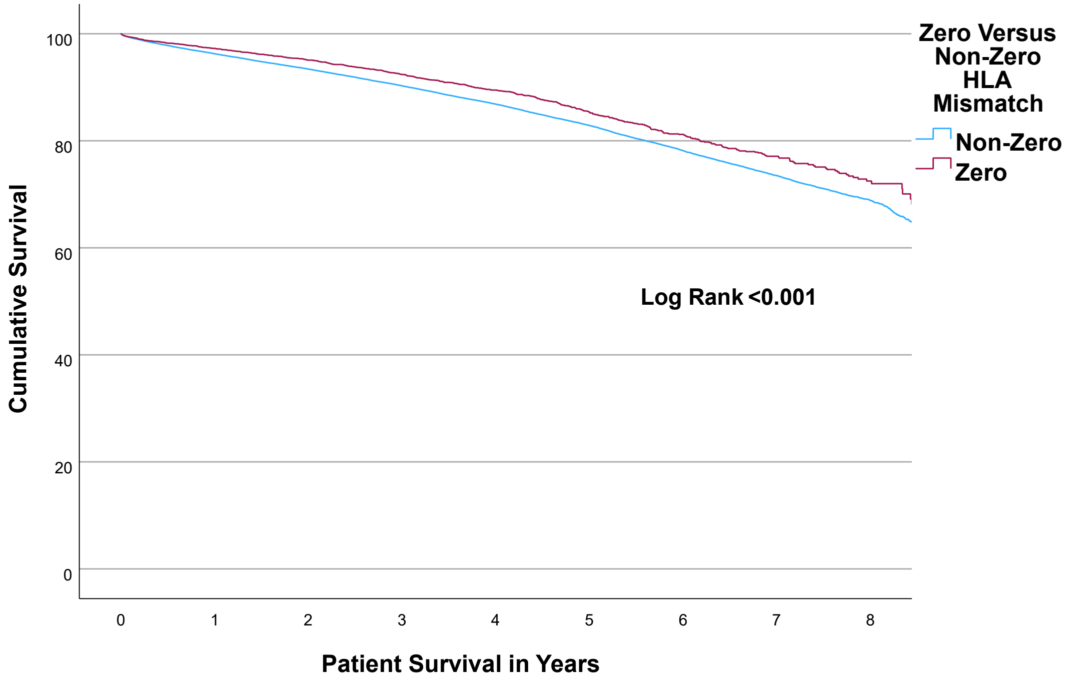
Zero Versus Non-Zero HLA Mismatch
Non-Zero
Zero
Log Rank <0.001
Cumulative Survival
100
80
60
40
20
0
0
1
2
3
4
5
6
7
8
Patient Survival in Years

Table 2A and B: Multiple Imputation Results for Graft and Patient Survival

Table 2A: Graft Survival

Imputation	Hazard Ratio of Graft Failure For Zero- HLA ABDR-Mismatch (Reference: Non-Zero HLA ABDR-Mismatch)			P value
	Hazard Ratio	95% Confidence Interval		
		Lower	Upper	
Original Data	0.898	0.834	0.967	0.004
Imputation 1	0.894	0.83	0.962	0.003
Imputation 2	0.894	0.83	0.963	0.003
Imputation 3	0.894	0.83	0.963	0.003
Imputation 4	0.894	0.83	0.963	0.003
Imputation 5	0.894	0.83	0.962	0.003
Pooled	0.894	0.83	0.962	0.003
Other Covariates in Model: Recipient Age, Race/Ethnicity, Gender, Cause of ESRD, CPRA, Duration of Dialysis Prior to Transplant, Previous Transplant, ABO Blood Type, Donor KDPI, Race, Gender, ABO Blood Type, and Cold Ischemia Time				

Table 2B: Patient Survival

Imputation	Hazard Ratio of Death For Zero- HLA ABDR- Mismatch (Reference: Non-Zero HLA ABDR-Mismatch)			P value
	Hazard Ratio	95% Confidence Interval		
		Lower	Upper	
Original Data	0.947	0.865	1.037	0.239
Imputation 1	0.944	0.862	1.033	0.211
Imputation 2	0.944	0.862	1.034	0.213
Imputation 3	0.944	0.862	1.033	0.212
Imputation 4	0.944	0.862	1.034	0.213
Imputation 5	0.944	0.862	1.033	0.212
Pooled	0.944	0.862	1.033	0.212
Other Covariates in Model: Recipient Age, Race/Ethnicity, Gender, Cause of ESRD, CPRA, Duration of Dialysis Before Transplant, Previous Transplant, ABO Blood Type, Donor KDPI, Race, Gender, ABO Blood Type, and Cold Ischemia Time				

In both patient and graft survival, the hazard ratios decreased slightly in the multiple imputation results, but the fundamental conclusion that zero HLA ABDR-mismatch recipients had a small graft survival benefit, but no patient survival benefit remained.

Subgroups Analysis of Graft and Patient Survival for recipient race/ethnicity and recipient ABO

Table 3A. Subgroup analysis of Zero HLA ABDR-mismatch compared to Non-Zero HLA ABDR mismatch for graft survival by recipient SRTR race/ethnicity for White, Black, and Latino recipients.

	Hazard Ratio of Graft Survival			
Race/Ethnicity Group	Hazard Ratio	95% C.I.		p value
		Lower	Upper	
White	0.880	0.801	0.967	0.008
Black	0.928	0.760	1.134	0.464
Latino	0.929	0.796	1.132	0.561
Covariates in Model: Age, Gender, Cause of Renal Disease, Previous Transplant, Duration of Dialysis, ABO Type, Cold Ischemia Time, KDPI, Donor ABO Type, Donor SRTR Race, and Donor Gender				

A graft-survival benefit was observed only among White recipients. For Black and Latino recipients, there was a trend toward better graft survival, but it did not reach statistical significance.

Table 3B. Subgroup analysis of Zero HLA ABDR-mismatch compared to Non-Zero HLA ABDR mismatch for patient survival by recipient SRTR race/ethnicity for White, Black, and Latino recipients.

	Hazard Ratio of Patient Survival			
Race/Ethnicity Group	Hazard Ratio	95% C.I.		p value
		Lower	Upper	
White	0.934	0.836	1.044	0.232
Black	0.928	0.710	1.213	0.584
Latino	1.000	0.799	1.252	0.999
Covariates in Model: Age, Gender, Cause of Renal Disease, Previous Transplant, Duration of Dialysis, ABO Type, Cold Ischemia Time, KDPI, Donor ABO type, Donor SRTR Race, and Donor Gender				

None of the SRTR racial and ethnic groups demonstrated a survival advantage.

Table 4A. Subgroup analysis of Zero HLA ABDR-mismatch compared to Non-Zero HLA ABDR mismatch for graft survival by recipient ABO type

	Hazard Ratio of Graft Survival			
Recipient ABO Type	Hazard Ratio	95% C.I.		p value
		Lower	Upper	
A	0.948	0.842	1.067	0.373
AB	0.865	0.559	1.339	0.516
B*	0.732	0.562	0.952	0.030
O	0.901	0.811	1.000	0.051
Covariates in Model: Age, Gender, SRTR Race/Ethnicity, Cause of Renal Disease, Previous Transplant, Duration of Dialysis, Cold Ischemia Time, KDPI, Donor ABO type, Donor SRTR Race, and Donor Gender				

*Blood type B recipients showed a graft-survival benefit for a Zero HLA ABDR mismatch compared with a Non-Zero HLA ABDR mismatch. Blood type O recipients approached significance, whereas A and AB showed no graft-survival benefit with a Zero HLA ABDR mismatch.

Table 4B. Subgroup analysis of Zero HLA ABDR-mismatch compared to Non-Zero HLA ABDR mismatch for patient survival by recipient ABO type

	Hazard Ratio of Patient Survival			
Recipient ABO Type	Hazard Ratio	95% C.I.		p value
		Lower	Upper	
A	1.022	0.887	1.178	0.794
AB	0.616	0.326	1.165	0.136
B*	0.692	0.497	0.964	0.030
O	0.976	0.859	1.110	0.716
Covariates in Model: Age, Gender, SRTR Race/Ethnicity, Cause of Renal Disease, Previous Transplant, Duration of Dialysis, Cold Ischemia Time, KDPI, Donor ABO type, Donor SRTR Race, and Donor Gender				

A, AB, and O recipient blood types showed no patient survival benefit of Zero HLA ABDR mismatch recipients compared to Non-Zero HLA ABDR mismatch recipients. *Patient survival was better in B recipients with a Zero HLA ABDR mismatch.
